# Exploratory Analysis of Association of Nightly Fasting and Sleep Durations with Colorectal Cancer Risk in Chinese Community-Dwelling Older Adults: A Cross-Sectional Study

**DOI:** 10.3390/nu18050861

**Published:** 2026-03-07

**Authors:** Peiqi Huang, Boyan Zeng, Sicheng Li, Ke Zhang, Chunhao Li, Yingru Liang, Bingyu Liuzhang, Xiaoli Wu, Shaohua Xie, Yan Li, Bo Zhang

**Affiliations:** 1 Food Safety and Health Research Center, School of Public Health, Southern Medical University, Guangzhou 510515, China; peiqihuang85@gmail.com (P.H.); zengbyan@foxmail.com (B.Z.); lsc19988292077@163.com (S.L.); zhangke20230120@126.com (K.Z.); ivylikesamericano@gmail.com (C.L.); eggyeggsy@smu.edu.cn (B.L.); wanwxl2022@126.com (X.W.); 2 Guangzhou Center for Disease Control and Prevention, Guangzhou Health Supervision Institute, Guangzhou 510440, China; wynniel@163.com; 3 School of Public Health, Fujian Medical University, Fuzhou 350122, China; shaohua.xie@ki.se; 4 Department of Molecular Medicine and Surgery, Karolinska Institutet, 17177 Stockholm, Sweden

**Keywords:** colorectal cancer, circadian rhythm, nightly fasting, sleep duration, cross-sectional study

## Abstract

**Objectives:** Disruptions in circadian-related behaviors are emerging as potential risk factors for gastrointestinal cancers. This study investigated the independent and joint associations of nightly fasting duration and sleep duration with the risk of colorectal cancer (CRC) among community-dwelling Chinese older adults. **Methods:** Participants were drawn from the Guangzhou CRC Screening Program, which used a questionnaire-based investigation, two separate fecal occult blood tests (FOBTs) for risk evaluation, and colonoscopy for high-risk individuals. Of the 347,297 people initially screened, 197,507 individuals were finally included after excluding 100,930 cases with missing eating/sleeping data or unknown/benign lesions via colonoscopy. Among the final sample, 351 CRC cases and 1384 precancerous lesions were diagnosed, while 195,772 individuals had negative results. Habitual times for dinner, breakfast, bedtime, and wake-up were used to define nightly fasting duration (dinner-to-breakfast) and nightly sleep duration (bedtime-to-wake). Multivariable logistic regression, subgroup analyses, and sensitivity analyses were performed to evaluate the associations. **Results:** In the fully adjusted models, each 1-h increment in nightly fasting duration was associated with a 9.5% (95% CI 1.039–1.153) higher risk of CRC, and the direct association was limited to individuals over 60 years (OR = 1.147, 95% CI 1.073–1.226), while each 1-h increment in nightly sleep duration was associated with a 15.2% (95% CI 0.806–0.893) lower risk of CRC. Consistently, earlier dinner, later breakfast and later bedtime were also associated with a higher CRC risk. **Conclusions:** In Guangzhou older residents, long nightly fasting duration was a risk factor for CRC, especially among individuals over 60 years old; while long nightly sleep duration was protective. These findings suggest that maintaining adequate sleep and optimizing the nightly fasting window may be viable lifestyle strategies for CRC prevention, emphasizing the need for tailored preventive measures for different age groups.

## 1. Introduction

Data from GLOBOCAN 2022 showed that colorectal cancer (CRC) was the second most common cause of cancer-related deaths worldwide, with more than 1,900,000 new CRC cases, accounting for 9.6% of all cancer cases [1]. The CRC burden is disproportionately distributed globally; around 28% of all new cases in 2019 occurred in China [2]. China experienced a sharp increase in CRC incidence from 1990 to 2019, with the age-standardized incidence rate rising from 12.52 per 100,000 to 30.55 per 100,000 [3], which parallels the ongoing rapid socioeconomic transition. In the United States, although the overall age-standardized CRC incidence rate has decreased by 46%, from 66.2 per 100,000 at its peak in 1985 to 35.7 per 100,000 in 2019, it still ranks second in cancer-related deaths and is the leading cause of death in men younger than 50 years [4]. The substantial rise in CRC burden among young adults aged 20–49 years (early-onset CRC), particularly in countries with a high socio-demographic index (SDI), imposes a new challenge in reducing CRC burden [5,6]. These geographical, temporal, and population variations in CRC incidences indicate that the modifiable factors play a key role in the malignant transformation of intestinal cells.

In the Global Burden of Disease Study, the summary contribution of ten risk factors (alcohol use, diet high in processed meat, diet high in red meat, diet low in calcium, diet low in fiber, diet low in milk, high body mass index, high fasting plasma glucose, low physical activity, and smoking) to disability-adjusted life years (DALY) of CRC was about 81%, which indicates that more unknown risk factors contribute to CRC burden [2]. The emerging risk factors, especially those that are increasingly common in younger generations, remain underexplored.

Circadian rhythm is the day–night variations in behavior, physiology, and metabolism that involve and impact every organ, which is autonomously regulated by the internal biological clock genes and synchronized with external cues [7]. Night shift work, which alters the timing of sleep, food intake, and activity, disrupting the circadian rhythm, is classified as “probably carcinogenic to humans” by the International Agency for Research on Cancer (IARC) based on evidence from human studies of breast and prostate cancer and their hormone-disrupting mechanisms [8]. However, the prevalence of circadian rhythm-disruptive lifestyles among persons without night shift work is increasing, and whether these behaviors increase the risk of non-hormone-dependent tumors, such as CRC, remains unclear.

Food is an important environmental time cue [9]. The times of eating and sleeping, i.e., the eating–fasting cycle and sleep–wake cycle, control the central-intestinal circadian synchronization [10]. The mismatch between eating and sleeping may induce circadian misalignment among organs, increasing the risk of CRC. Interest in fasting duration and meal timing has grown, yet most related studies still focus on time-restricted eating [11,12,13]. Some studies have suggested that a longer nightly fasting duration is associated with improved metabolic profiles and reduced cancer risk [14,15,16]. However, animal data indicated that prolonged fasting may promote CRC through elevated bile acids [17,18]. These mixed findings highlight the need for population-based research on habitual fasting patterns. And the timing of dinner and breakfast determines the nightly fasting duration. However, no unified standard defines “early” or “late” meals.

In this study, we included patients newly diagnosed with CRC or precancerous lesions and a non-CRC group from the CRC screening program in Guangzhou, China. We aimed to assess the habitual timing of dinner, bedtime at night, wake time in the morning, and breakfast time, in addition to nightly fasting duration and nightly sleep duration.

## 2. Materials and Methods

### 2.1. Study Design and Population

This cross-sectional study used data from the Guangzhou CRC Screening Program. Since 2015, the program has collected personal information and risk assessment questionnaires and has conducted CRC screening among permanent residents aged 50–74 years in Guangzhou. Recruitment was carried out through public media (newspapers and television) and supplemented by short message service reminders sent to the target age group. Participation in the screening was entirely voluntary. The two-stage screening strategy has been described previously [19,20]. Briefly, the CRC risk assessment questionnaire and fecal occult blood test (FOBT) were used to screen high-risk individuals in the first stage. The risk assessment questionnaire includes ID number, age, sex, weight and height, lifestyle (i.e., drinking, smoking), history of non-CRC cancer, history of polyps, family history of CRC in a first-degree relative, and intestinal symptoms in the last two years and history of specific intestinal diseases. The positive criteria of the risk assessment questionnaire are (1) history of CRC in first-degree relatives; (2) personal history of non-CRC; (3) personal history of intestinal polyps; and (4) two or more of the following: chronic constipation, chronic diarrhea, mucous bloody stool, history of adverse life events, history of chronic appendicitis or appendectomy, and history of chronic biliary disease or cholecystectomy. FOBTs were performed twice with at least one week’s intervals. Individuals with any positive results from the two tests were considered positive for the FOBT. The participants who were identified as positive in either the questionnaire or FOBT were considered high-risk individuals and were referred to undergo a colonoscopy in the second stage. In the first round of screening from January 2015 to December 2017, 347,297 participants with a unique ID number were enrolled in the CRC screening system.

**Figure 1:**
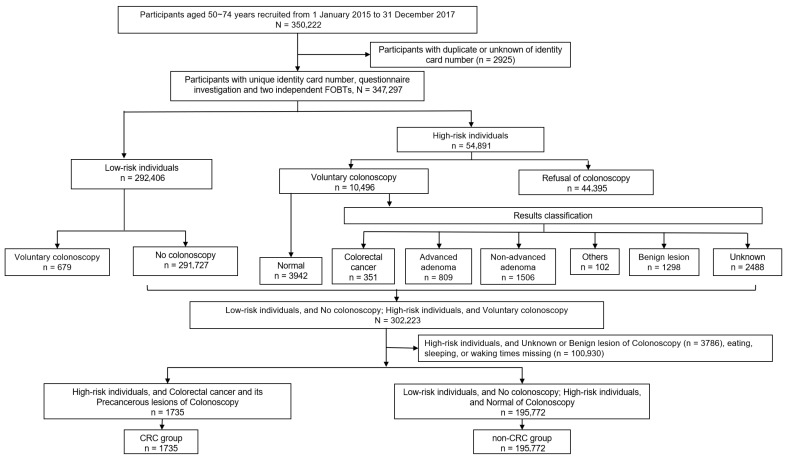
Flow chart of the subjects in the Guangzhou CRC Screening Program (2015–2017).

In the second stage of the two-stage screening strategy, participants with positive results from the first stage (either the risk assessment questionnaire or FOBT) were recommended to undergo colonoscopy. The overall data screening process is shown in Figure 1. We excluded 679 participants with negative screening results who nevertheless underwent colonoscopy due to gastrointestinal symptoms or personal preference. These examinations were not part of the structured screening program and did not affect the results. Among those who underwent colonoscopy, the results were classified into CRC (*n* = 351), precancerous lesions (*n* = 2315), and unknown or benign results (*n* = 3786). After excluding the participants without timing information of eating, waking and sleeping (*n* = 100,930), the CRC group comprised participants diagnosed with CRC (*n* = 227) and its precancerous lesions (*n* = 1508). Participants with negative screening results at stage I, as well as those with positive screening results at stage I but normal colonoscopy results, were classified as the non-CRC group (*n* = 195,772).

Ultimately, the CRC group (*n* = 1735) and the non-CRC group (*n* = 195,772) were determined based on the two-stage screening strategy.

### 2.2. Ascertainment of CRC and Precancerous Lesions

For the ascertainment of the CRC group, we strictly followed the relevant medical diagnostic criteria and procedures to ensure the precision and reliability of the diagnosis. The CRC lesions identified during colonoscopy included cancer (*n* = 351), advanced adenomas (*n* = 809), non-advanced adenomas (*n* = 1506), and non-adenomatous lesions (*n* = 102), such as sessile serrated polyps, and the latter three were considered precancerous. Both CRC and precancerous lesions were defined as CRC cases in the study.

The non-CRC cases in our study represented the negative individuals in the CRC screening program, including those who were identified as low-risk by the questionnaire and FOBT and did not undergo colonoscopy (*n* = 291,727), or those who underwent colonoscopy but had normal results (*n* = 3942).

### 2.3. Collection of Exposure Information and Confounding Factors

We collected the habitual times of dinner, bedtime at night, wake time in the morning, and breakfast time. We also calculated nightly fasting duration, nightly sleep duration, dinner-to-sleep interval, and wake-up-to-breakfast interval. Participants without this information (*n* = 100,930) were excluded. All exposure variables were obtained through structured self-reported questionnaires, which may introduce recall bias. Mealtimes and fasting-related measures were coarse because the questionnaire did not capture information on late-night snacks, intra-individual variability in mealtimes, or sleep quality. Therefore, the estimated fasting duration—calculated as the time from dinner to breakfast—may overestimate the actual fasting period if snack consumption occurred during the night.

We also included confounding factors such as age, sex, marital status, educational level, body mass index (BMI; calculated using weight and height), diabetes history, and smoking and drinking history. As the routine screening protocol did not include detailed assessments of diet quality, physical activity, or medication use, these variables were not part of the adjusted models.

### 2.4. Statistical Analysis

Given the cross-sectional design of the study, the analyses assessed associations rather than causal relationships. We used logistic regression analysis to analyze the association between nightly fasting and nightly sleep durations and the risk of CRC. We summarized continuous variables using mean and standard deviation (SD). To identify potential nonlinear relationships, we divided the nightly fasting duration, nightly sleep duration, and mealtimes into groups based on the median value of the samples. Odds ratios (ORs) were calculated after adjusting for potential confounding factors (Model 1) and nightly fasting duration (Model 2). All statistical analyses were performed using R version 4.4.1 (R Foundation for Statistical Computing, Vienna, Austria). This is an exploratory study focused on hypothesis generation. Therefore, we did not apply formal adjustments for multiple testing, such as Bonferroni correction, to avoid missing potentially important associations. We interpret our findings based on the consistency of patterns and 95% confidence intervals, treating them as descriptive indicators rather than definitive tests of significance.

To handle the collinearity between nightly fasting duration and nightly sleep duration, we conducted a sensitivity analysis. We included both nightly fasting duration and nightly sleep duration simultaneously in the model to evaluate their independent effects. We further calculated the active period of fasting duration (nightly fasting duration minus sleep duration) and the ratio of nightly sleep duration to fasting duration and assessed their relationships with CRC risk. Additionally, we performed a subgroup analysis by age and sex and a sensitivity analysis by excluding cancer lesions to test the robustness of the associations.

## 3. Results

### 3.1. Participant Characteristics

Table 1 shows the main characteristics of the study population, who were divided into the CRC and non-CRC groups. In this cross-sectional study, 197,507 participants were included. The CRC group included 1735 participants, 830 women (47.8%) and 905 men (52.2%), with a mean age of 62.30 years (SD = 6.16). The non-CRC group consisted of 195,772 participants, 125,879 women (64.3%) and 69,893 men (35.7%), with a mean age of 61.42 years (SD = 6.44). The CRC group included individuals with precancerous lesions and cancer. Table 1 shows that the CRC group had a mean nightly fasting duration of 13.02 ± 0.91 h, nightly sleep duration of 7.87 ± 0.98 h, dinner-to-sleep interval of 4.00 ± 0.94 h, and a wake-up-to-breakfast interval of 1.16 ± 0.71 h. The non-CRC group had a nightly fasting duration of 12.92 ± 0.91 h, nightly sleep duration of 7.98 ± 0.93 h, dinner-to-sleep interval of 3.81 ± 0.92 h, and wake-up-to-breakfast interval of 1.13 ± 0.7 h (Table 1). Supplementary Table S1 data show that the first-stage positive rate (8.7%), average age (≈62 years), and BMI (23.5 ± 3.0 kg/m^2^) in the exclusion group were similar to those in the inclusion group (8.9%, 61.4 years, 23.5 ± 3.4 kg/m^2^). Although the *p*-value of the disease history was <0.001, these differences were mainly due to the systematic absence of questionnaire data (NA ≈ 96% in the exclusion group). This supports selection bias as non-dissimilarity and has a limited impact on effect estimation (Table S1).

### 3.2. Association Between Nightly Fasting Duration and CRC Risk

Multivariable-adjusted associations between nightly fasting duration and CRC risk are presented in Table 2 and Table 3. After adjusting for age, BMI, sex, marital status, education, smoking status, alcohol intake frequency, and history of diabetes, each 1-h increment in nightly fasting duration per day was associated with a 9.5% (95% CI 1.039–1.153) higher risk of CRC. To examine the robustness of the results, we also conducted logistic regression analysis for the association between CRC risk and dinner-to-bed interval, wake-up-to-breakfast interval, and mealtimes. As continuous variables, each 1-h increment in dinner-to-bed interval per day was associated with a 24.2% (95% CI 1.171–1.318) higher risk of CRC, while the association for the wake-up-to-breakfast interval was characterized by an OR of 0.998 (95% CI 0.932–1.067). The results on mealtimes showed that each 1-h increment in delayed breakfast time per day was associated with a 6.5% (95% CI 1.001–1.132) higher risk of CRC, while each 1-h increment in delayed dinner time per day was associated with a 10.2% (95% CI 0.843–0.961) lower risk of CRC. The results of categorical variable analysis were consistent with those of continuous variable analysis. These results supported that longer nightly fasting duration was associated with an increased risk of CRC (Table 2 and Table 3).

To explore the potential explanations for this discrepancy, we conducted subgroup analysis by age. In the age subgroup analysis, the association among individuals younger than 60 yielded an OR of 1061 (95% CI 0.935–1.103), while each 1-h increment in nightly fasting duration was associated with a 14.7% (95% CI 1.073–1.226) higher risk of CRC in individuals over 60 years. In individuals over 60 years, a nightly fasting duration of 12–14 h yielded an OR of 1.205 (95% CI 0.907–1.643) compared with the reference group (<12 h), and the ≥14 h group was associated with a 51.0% (95% CI 1.112–2.094) higher risk of CRC. The association between dinner-to-bed interval, wake-up-to-breakfast interval, and CRC in this age subgroup was similar to that observed in the overall analysis. The association between mealtimes and CRC in individuals over 60 years was also similar to that in the CRC group, while there was no directional association observed in individuals younger than 60 years. In individuals over 60, each 1-h increment in breakfast time per day was associated with a 10.1% (95% CI 1.019–1.190) higher risk, and dinner time per day was associated with a 14% (95% CI 0.797–0.933) lower risk of CRC (Table 4 and Table 5).

We also conducted subgroup analysis to examine whether the associations were modified by sex. In the sex subgroup analysis, continuous analysis revealed that each 1-h increment in nightly fasting duration per day was associated with a 10.6% (95% CI 1.030–1.187) higher risk of CRC in men and an 8.3% (95% CI 1.003–1.168) higher risk in women. Consistent results were observed in the dinner-to-bed interval and dinner time. The results of categorical variable analysis were consistent with those of continuous variable analysis. Both were consistent with the CRC group and supported that the longer nightly fasting duration was an increased risk factor for CRC. In multivariable model 1 after adjusting for age, BMI, sex, marital status, education, smoking status, alcohol intake frequency, and history of diabetes, each 1-h increment in the wake-up-to-breakfast interval was associated with a 10.7% (95% CI 1.009–1.211) higher risk of CRC in men, but the OR for CRC in women was 0.987 (95% CI 0.999–1.172). With respect to breakfast time, no clear directional associations were observed in either men or women (Tables S2 and S3). We also performed age-stratified analyses by sex. The analyses of nightly fasting duration and mealtimes showed broadly consistent association patterns between men and women, with no opposite or meaningfully divergent directions observed in any sex-specific age subgroup (Tables S11–S14).

We found that the time interval of the nightly fasting duration in our individuals was rather unusual: 58.5% had dinner time at or before 18:30, and 63.7% had breakfast time at or before 7:30. Consequently, the mean nightly fasting duration reached 13 h, with 20% of the individuals fasting for 14 h or longer.

In conclusion, the nightly fasting duration in this study is rather special, and there is an association between nightly fasting duration and CRC risk, with longer nightly fasting duration being a risk factor, especially in individuals over 60.

### 3.3. Association Between Nightly Sleep Duration and CRC Risk

Multivariable-adjusted associations between nightly sleep duration and CRC risk are presented in Table 2 and Table 3. After adjusting for potential confounding factors and nightly fasting duration, each 1-h increment in nightly sleep duration was associated with a 15.2% (95% CI, 0.806–0.893) lower risk of CRC. In multivariable model 2, which was further adjusted for fasting time based on model 1, individuals with a nightly sleep duration of ≥9 h had a 16.5% (95% CI 0.740–0.941) lower risk of CRC than did those with <9 h. To assess the robustness of the results, we also conducted logistic regression analyses at the two time points of wake time and bedtime, and only each 1-h increment in bedtime was associated with a 17.2% (95% CI 1.100–1.248) higher risk of CRC. The results of categorical variable analysis were consistent with those of continuous variable analysis. These results supported that longer nightly sleep duration was a protective factor for CRC (Table 2 and Table 3).

We also conducted subgroup analysis to explore whether the associations were modified by age and sex. In the age subgroup analysis, the association between nightly sleep duration and CRC was the same as that in the CRC group. In the multivariable model 2, each 1-h increment in nightly sleep duration was associated with a 14.6% (95% CI 0.786–0.928) lower risk of CRC in individuals less than 60 years and a 6.8% (95% CI 0.874–0.994) lower risk of CRC in individuals over 60 years. However, among individuals younger than 60 years, a nightly sleep duration of ≥9 h was characterized by an OR of 0.910 (95% CI 0.743–1.116) compared with the reference group (<9 h). The association between wake time, bedtime and CRC in the age subgroup was the same as that in the CRC group (Table 4 and Table 5). Furthermore, the age- and sex-stratified analyses of precancerous lesions yielded similar results. Both the age-stratified (Tables S6 and S8) and sex-stratified (Tables S7 and S9) analyses demonstrated patterns consistent with the CRC group, further supporting the robustness of the findings.

In the sex-stratified analysis, continuous analysis revealed that each 1-h increment in nightly sleep duration was associated with a 14.5% (95% CI 0.797–0.917) lower risk of CRC in men and a 10.6% (95% CI 0.828–0.966) lower risk in women. The results of the wake time and bedtime were consistent with sex. After multivariable adjustments, the associations for wake time yielded an OR of 0.917 in men (95% CI 0.794–1.059) and 0.983 in women (95% CI 0.896–1.078), while each 1 h in bedtime was associated with a 17.1% (95% CI 1.076–1.275) higher risk of CRC in men and a 17.3% (95% CI 1.068–1.289) higher risk in women. The results of categorical variable analysis were consistent with those of continuous variable analysis. Both were consistent with the CRC group and supported that the longer nightly sleep duration was a protective factor for CRC (Tables S2 and S3). We also performed age-stratified analyses in both sex subgroups. The analyses of nightly sleep duration, bedtime and wake time showed broadly consistent association patterns between men and women, with no opposite or meaningfully divergent directions observed in any sex-specific age subgroup (Tables S11–S14).

In conclusion, there is an association between nightly sleep duration and CRC risk, with longer nightly sleep duration being a protective factor, with consistent results by age and sex.

### 3.4. Sensitivity Analysis

To examine the robustness of the results in the main analysis, we conducted a sensitivity analysis on the precancerous lesion group in the CRC group. After multivariable adjustments, each 1-h increment in nightly fasting duration was associated with a 9.8% (95% CI 1.039–1.160) higher risk of CRC in the precancerous lesion group. Furthermore, each 1-h increment in the dinner-to-bed interval was associated with a 15.1% (95% CI 1.080–1.228) higher risk of CRC, and each 1-h increment in dinner time was associated with an 11.5% (95% CI 0.829–0.950) lower risk of CRC, while the association was characterized by an OR of 0.998 (95% CI 0.964–1.114) in the wake-up-to-breakfast interval. The results of categorical variable analysis were consistent with those of continuous variable analysis. Both were consistent with the CRC group. When stratified by precancerous lesions, the association for breakfast time yielded an OR of 1.063 (95% CI 0.995–1.134). Notably, within the precancerous lesions subgroup, having breakfast after 7:30 was associated with a more pronounced estimate (OR = 1.165, 95% CI 1.049–1.292) compared to having breakfast before 7:30. In addition, the results of the continuous analysis indicated that longer nightly sleep duration was a protective factor for CRC. Each 1-h increment in nightly sleep duration was associated with a 12.1% (95% CI 0.831–0.929) lower risk of CRC. Furthermore, each 1-h increment in bedtime was associated with a 15.2% (95% CI 1.076–1.232) higher risk of CRC, while the association was characterized by an OR of 0.942 (95% CI 0.886–1.002) in wake time. The results of categorical variable analysis were consistent with those of continuous variable analysis. Both were consistent with the CRC group (Tables S4 and S5).

As nightly fasting duration included nightly sleep duration, the Pearson correlation coefficient between nightly fasting and nightly sleep durations was 0.260 (*p* < 0.001), indicating a weak correlation. We performed univariate and multivariate sensitivity analyses to assess their independent effects on CRC risk. We further calculated the “active period of nightly fasting duration” (nightly fasting duration minus nightly sleep duration). Logistic regression analysis showed that each 1-h increment in the active period of nightly fasting duration was associated with a 14.3% (95% CI 1.096–1.191) higher risk for CRC, supporting the independent role of the fasting duration. Additionally, we analyzed the ratio of nightly sleep duration to nightly fasting duration in relation to CRC risk. Logistic regression analysis revealed that the ratio was inversely associated with the risk of CRC (OR = 0.130, 95% CI: 0.068–0.246) (Table S10).

The results of the sensitivity analysis indicated that the effects of nightly fasting duration and nightly sleep duration on the risk of CRC were independent and robust.

## 4. Discussion

In this cross-sectional study, using data from the Guangzhou Colorectal Cancer Screening Project, we found that longer nightly fasting duration and shorter nightly sleep duration were associated with higher CRC risk.

### 4.1. Interpretation of the Association Between Nightly Fasting Duration and CRC Risk

To our knowledge, this is the first study reporting that long nightly fasting duration was a risk factor for CRC among community-dwelling Chinese. However, when conducting subgroup analysis by age, this direct association was evident only among individuals over 60 years of age. The results were robust, no matter that the nightly fasting duration was treated as a continuous or categorical variable. Furthermore, when mealtimes were analyzed, the effects of longer nightly fasting duration might be, at least partially, attributed to early dinner time.

Although there is considerable evidence supporting that nightly fasting duration has benefits on weight loss and cardiometabolic risk factors [21] and may enhance the efficacy of various cancer therapies [22]. The relationship between nightly fasting duration and health is inconclusive. Existing research does not consistently support prolonged fasting duration as a protective factor against diseases. A cross-sectional study [23] found that among elderly people in the community, prolonged nightly fasting duration (≥12 h) was associated with lower high-density lipoprotein cholesterol, indicating that it did not improve cardiovascular health in the elderly. Instead, it may be related to dyslipidemia and electrolyte imbalance. It indicates that the long extension between two meals (dinner to breakfast interval) is not beneficial to the elderly. Moreover, a cohort study [24] also found that long nightly fasting duration had no protective effect on type 2 diabetes (T2D). Instead, having breakfast later was significantly associated with increased T2D incidence.

Our finding, if reproducible, has some biological plausibility. While our study did not quantify biological markers directly, the observed association is supported by biological plausibility derived from experimental models. For instance, a recent study in mice suggested that post-fast feeding helps to activate intestinal stem cells, which are a source of precancerous cells [25]. Repeated fasting in mice has deleterious effects on the levels of B cells in intestinal Peyer’s patches, and their survival is drastically reduced and restored by re-feeding; thus, the normal immune response to oral immunization is attenuated by fasting [26]. Another study [27] found that fasting and refeeding disrupted the normal renewal rhythm of monocytes, released a batch of aged immune cells, and increased the risk of infection and inflammatory responses. Specifically, the numbers of various immune cells in the lamina propria of the small and large intestines were altered, potentially exacerbating inflammatory fluctuations. Monocytes may also promote age-related inflammation, and immune function declines with age [28]. These age-related inflammatory changes can impair intestinal barrier function, leading to excessive immune activation and thereby promoting CRC development [29,30,31]. Adults over 60 years of age are prone to circadian rhythm asynchrony [32], which is closely related to changes in the abundance and function of the gut microbiota. These changes in the structure and function of the microbiota further induce adverse health outcomes, such as CRC and cardiovascular disease [33].

At the same time, we found that the time interval of nightly fasting durations in our individuals was unique: 58.5% had dinner at or before 18:30, and 63.7% finished breakfast at or before 07:30. Consequently, the mean nightly fasting duration reached 13 h, with 20% of the individuals fasting for 14 h or longer at night. However, most previous studies set dinner at 20:00–21:00 and breakfast at 08:00–09:00, with the fasting duration mostly being 11 to 13 h [24,34,35]. This is a substantial difference in the length of nightly fasting duration among these studies. Furthermore, the starting and ending time points of nightly fasting, which reflect the coherence between eating behavior and endogenous circadian rhythm, have independent or joint effects with fasting duration on health [36,37,38]. A previous study has found that the disorder of the biological clock can drive the occurrence of CRC by affecting the intestinal microbiota and intestinal permeability [39]. There is also epidemiological evidence that circadian rhythm disorders can have a negative impact on the microbiota in the gastrointestinal tract and may lead to the development of metabolic syndrome and cancer [40].

The interpretation of nightly fasting duration should consider cultural dietary habits and unmeasured lifestyle factors [41,42,43]. In this study, an early dinner resulted in longer fasting duration. This pattern may differ from populations with later meal times. Diet quality and physical activity were not measured, although both are closely linked to meal timing and influence colorectal cancer risk. These missing factors may partly explain the observed associations. Recent characterizations of CRC patients also show that unhealthy diets, low physical activity, and irregular eating patterns often occur together [44]. These issues limit the general applicability of our findings. Therefore, the findings of this study are only applicable to the elderly Chinese population who “eat dinner and breakfast early”, and should not be directly extrapolated to other populations with later dinner habits. In the future, more comparisons should be made of the health effects of the same fasting duration but different eating windows.

In summary, we identified long nightly fasting as a risk factor for CRC in the elderly. This adverse effect likely stems from inflammation driven by the fasting-refeeding cycle and the specific “early-dinner” pattern. Therefore, fasting recommendations should not be universal but must consider age and specific eating windows.

### 4.2. Interpretation of the Association Between Nightly Sleep Duration and CRC Risk

Our study found that longer nightly sleep duration, whether as a continuous or categorical variable, was a protective factor for CRC. The reverse association was further supported by the later bedtime as a risk factor for CRC. Our finding aligns with the existing studies. A study of Chinese middle-aged and older adults found that short sleep may increase cancer risk, especially for digestive and respiratory cancers, while longer sleep showed no significant link [45]. Similarly to our study, the study population also included middle-aged and older adults in China. A case–control study of older adults in Vietnam also found that short sleep duration is a risk factor for colorectal adenoma, an important precursor of CRC [46]. Additionally, a study of the American population found that short sleep duration was associated with an increased risk of colorectal adenoma [47]. Further studies are needed to identify the optimal nightly sleep duration range to reduce CRC risk. The association between sleep duration and CRC may be shaped by underlying circadian rhythms.

Sleep behaviors, including sleep duration and timing, are outward expressions of the circadian system [48]. Various studies explored the relationship between nightly sleep duration and CRC risk, with most focusing on the impact of circadian rhythms. A case–control study in Pakistan found that irregular sleep patterns may increase the risk of CRC [49]. Another study found that the circadian clock controls the genes involved in cell proliferation, and maintaining its rhythmicity is essential for controlling the occurrence and development of tumors. Disruption of circadian rhythms leads to abnormal expression of circadian genes, which are closely associated with the development and progression of CRC [50]. An animal experiment revealed that disruption of the circadian clock influences the loss of heterozygosity in the Apc gene, thereby hyperactivating the Wnt signaling pathway and accelerating the progression of CRC [51]. Further studies are required to provide more insights into understanding and preventing CRC. Future studies could further focus on the regulatory mechanisms of circadian rhythms and explore how optimizing sleep patterns may reduce CRC risk.

### 4.3. Sex Differences in CRC Incidence and Compliance

The associations observed in this study were directionally consistent in men and women. However, the sex distribution of confirmed CRC cases requires clarification. First, the previous literature has shown that males are associated with an increased risk of CRC [52], and there is gender bimorphism in CRC tumor biology [53]. Furthermore, across most age groups, the incidence of CRC is higher in men than in women, but the difference is most marked between the ages of 55 and 84 years [54]. Therefore, even though the overall screening population had a higher proportion of women, the higher proportion of men among confirmed cases is consistent with the expected epidemiological pattern. Second, colonoscopy compliance was substantially lower among women in this program. Among participants who completed primary screening but did not undergo colonoscopy, 63% were women and 37% were men. This lower adherence resulted in more women discontinuing the diagnostic pathway and likely contributed to the higher proportion of men among diagnosed cases. Finally, missing questionnaire data among men was limited (38%) and is unlikely to have introduced systematic bias. Taken together, these factors explain the observed sex distribution and indicate that the findings are not driven by sex-related selection bias.

### 4.4. Strengths and Limitations

As we known it is the first study collecting nightly eating and sleeping time simultaneously in community-dwelling residents who underwent CRC screening. Longer nightly fasting duration was first discovered as a risk factor for CRC in older adults. We further verified the correlation between nightly fasting duration and nightly sleep duration and the risk of CRC by analyzing four time points related to the circadian rhythm. The large and representative sample size supported stable estimates and informative subgroup analyses. Sensitivity analysis using precancerous lesions can partially reduce the effects of reverse causation. Furthermore, all information was derived from questionnaires validated through pilot testing and expert consultation, thus enhancing the reliability of the data.

This study also had limitations. First, local dietary habits, such as early dinner and late breakfast, may influence the interpretation of nightly fasting duration. These habits led to a relatively long fasting interval in our population and may limit the generalizability of the findings. In addition, because late-night snacking was not captured in the data collection, fasting duration may have been slightly overestimated, especially among individuals who eat dinner early. Second, we failed to collect some potential confounding factors, including meal duration, late-night snacking, diet quality and socioeconomic status. These unmeasured variables may limit the strength and generalizability of our conclusions. Moreover, the data at the four time points were recorded at a single time, and the data were relatively coarse. Third, as a cross-sectional study, the temporal sequence remains unclear, and causality cannot be established. We lack biological samples (such as blood or tissue) to verify the mechanisms. Therefore, the biological mechanisms discussed in this paper are still speculative and need to be verified in future translational research. Fourth, low compliance with colonoscopy might lead to selection bias. Broader dietary and sleep-related behavior patterns may also affect fasting duration and warrant further investigation.

Our results suggest that meal timing and sleep patterns may have implications for personalized lifestyle approaches in CRC prevention, but these possibilities require confirmation in longitudinal research. Future prospective studies using objective assessments (e.g., actigraphy and real-time dietary tracking) are needed to confirm our findings and further elucidate the underlying causal pathways.

## 5. Conclusions

Among Guangzhou residents, long nightly fasting duration was a risk factor for CRC, especially among individuals over 60 years of age, while long nightly sleep duration was a protective factor for CRC. While the findings provide new insights into nightly fasting and sleep duration and circadian-related behaviors in a CRC screening population, they should be interpreted with caution due to the study’s limitations. Although this is a preliminary study, the results may be heuristic for future research to explore the mechanisms underlying these associations.

## Figures and Tables

**Table 1:** Characteristics of the Study Participants.

Variable	CRC (*n* = 1735)	Non-CRC (*n* = 195,772)	*p*
Age, y	62.30 ± 6.16	61.42 ± 6.44	<0.001
Sex, %			<0.001
Male	905 (52.2)	69,893 (35.7)	
Female	830 (47.8)	125,879 (64.3)	
BMI, kg/m^2^	23.67 ± 3.38	23.52 ± 3.35	0.056
Marital status, %			0.880
Married	1635 (94.2)	184,633 (94.3)	
Other	100 (5.8)	11,139 (5.7)	
Education, %			<0.001
Illiterate	39 (2.3)	7406 (3.8)	
Primary school	432 (24.9)	70,993 (36.3)	
Secondary school	1005 (57.9)	102,105 (52.2)	
College	241 (13.9)	14,677 (7.5)	
Postgraduate	18 (1.0)	580 (0.2)	
NA	0 (0.0)	11 (0.0)	
Diabetes, %			0.162
No	1515 (87.3)	168,489 (86.1)	
Yes	180 (10.4)	21,388 (10.9)	
NA	40 (2.3)	5895 (3.0)	
Smoking, %			<0.001
Never	1279 (73.7)	164,264 (83.9)	
Current	301 (17.3)	21,775 (11.1)	
Quit	155 (8.9)	9705 (5.0)	
NA	0 (0.0)	28 (0.0)	
Alcohol, %			<0.001
Hardly drink	1460 (84.1)	178,013 (90.9)	
Yes, but not every month	117 (6.7)	8427 (4.3)	
Yes, but not every week	75 (4.3)	4586 (2.3)	
Yes, more than once a day	83 (4.8)	4695 (2.4)	
NA	0 (0.0)	51 (0.0)	
Nightly fasting duration, h	13.02 ± 0.91	12.92 ± 0.91	<0.001
Nightly sleep duration, h	7.87 ± 0.98	7.98 ± 0.93	<0.001
Dinner-to-bed interval, h	4.00 ± 0.94	3.81 ± 0.92	<0.001
Wake-to-breakfast interval, h	1.16 ± 0.71	1.13 ± 0.72	0.178

BMI indicates body mass index. Data were mean ± SD or number (percentage). NA: Not available (non-response).

**Table 2:** Associations of nightly fasting and sleep duration with CRC risk, *n* = 197,507.

Variable	Precancerous and Cancer Lesions	
	*n* CRC/Non-CRC	OR (95% CI)
Nightly fasting duration 1		
<12	78/11,603	1 (Ref.)
12~14	1254/145,025	1.213 (0.971, 1.539)
≥14	403/39,144	1.389 (1.093, 1.788)
Continuous (1 h incr)	1735/195,772	1.095 (1.039, 1.153)
Nightly Sleep duration 2		
<9	1382/150,037	1 (Ref.)
≥9	353/45,735	0.835 (0.740, 0.941)
Continuous (1 h incr)	1735/195,772	0.848 (0.806, 0.893)
Dinner-to-bed interval 2		
<4	548/76,225	1 (Ref.)
≥4	1187/119,547	1.196 (1.072, 1.336)
Continuous (1 h incr)	1735/195,772	1.242 (1.171, 1.318)
Wake-to-breakfast interval 2		
<1	421/46,477	1 (Ref.)
≥1	1314/149,295	0.967 (0.867, 1.083)
Continuous (1 h incr)	1735/195,772	0.998 (0.932, 1.067)

1; model adjusted for age, BMI, marital status, education, smoking, alcohol, diabetes. 2; model adjusted for fasting time in addition.

**Table 3:** Associations of mealtimes, bedtime and wake time with CRC risk. *n* = 197,507.

Variable	*n* CRC/Non-CRC	OR (95% CI)
Breakfast time 1		
≤7:30	1031/124,744	1.00
>7:30	704/71,028	1.160 (1.052, 1.278)
Continuous (1 h incr)	1735/195,772	1.065 (1.001, 1.132)
Dinner time 1		
≤18:30	1055/114,475	1.00
>18:30	680/81,297	0.889 (0.806, 0.981)
Continuous (1 h incr)	1735/195,772	0.898 (0.843, 0.961)
Wake time 2		
≤6:30	1162/134,206	1.00
>6:30	573/61,566	0.929 (0.836, 1.031)
Continuous (1 h incr)	1735/195,772	0.942 (0.886, 1.002)
Bedtime 2		
≤22:30	1072/134,297	1.00
>22:30	663/61,475	1.241 (1.122, 1.371)
Continuous (1 h incr)	1735/195,772	1.172 (1.100, 1.248)

1; ORs were adjusted for age, sex, BMI, marital status, education, smoking, alcohol, diabetes, breakfast time or dinner time. 2; ORs were adjusted for age, sex, BMI, marital status, education, smoking, alcohol, diabetes, wake time or bedtime. Abbreviations: CI, confidence interval; OR, odds ratio.

**Table 4:** Subgroup analysis of nightly fasting and sleep duration with CRC risk by age, *n* = 197,507.

Variable	Age			
	≤60		>60	
	*n* CRC/Non-CRC	OR (95% CI)	*n* CRC/Non-CRC	OR (95% CI)
Nightly fasting duration 1				
<12	31/5193	1 (Ref)	47/6410	1 (Ref.)
12~14	460/61,341	1.183 (0.835, 1.739)	794/83,684	1.205 (0.907, 1.643)
≥14	147/18,063	1.220 (0.838, 1.834)	256/21,081	1.510 (1.112, 2.094)
Continuous (1 h incr)	638/84,597	1.016 (0.935, 1.103)	1097/111,175	1.147 (1.073, 1.226)
Nightly sleep duration 2				
<9	507/65,331	1 (Ref)	875/84,706	1 (Ref.)
≥9	131/19,266	0.910 (0.743, 1.116)	222/26,469	0.795 (0.681, 0.923)
Continuous (1 h incr)	638/84,597	0.854 (0.786, 0.928)	1097/111,175	0.932 (0.874, 0.994)
Dinner-to-bed interval 2				
<4	181/30,377	1 (Ref)	367/45,848	1 (Ref.)
≥4	457/54,220	1.014 (0.886, 1.061)	730/65,327	1.173 (1.025, 1.344)
Continuous (1 h incr)	638/84,597	1.170 (1.075, 1.272)	1097/111,175	1.185 (1.108, 1.266)
Wake-to-breakfast interval 2				
<1	187/21,608	1 (Ref)	234/24,869	1 (Ref.)
≥1	451/62,989	0.858 (0.723, 1.024)	863/86,306	1.022 (0.884, 1.187)
Continuous (1 h incr)	638/84,597	1.035 (0.926, 1.152)	1097/111,175	1.011 (0.928 1.100)

1; model adjusted for BMI, marital status, education, smoking, alcohol, and diabetes. 2; model adjusted for fasting time in addition.

**Table 5:** Association of mealtimes, bedtime and wake time with CRC risk by age. *n* = 197,507.

Variable	Age			
	≤60		>60	
	*n* CRC/Non-CRC	OR (95% CI)	*n* CRC/Non-CRC	OR (95% CI)
Breakfast time 1				
≤7:30	364/51,950	1.00	667/72,794	1.00
>7:30	274/32,647	1.127 (0.961, 1.066)	430/38,381	1.161 (1.026, 1.312)
Continuous (1 h incr)	638/84,597	0.993 (0.898, 1.097)	1097/111,175	1.101 (1.019, 1.190)
Dinner time 1				
≤18:30	350/47,487	1.00	705/66,988	1.00
>18:30	288/37,110	1.033 (0.882, 1.210)	392/44,187	0.804 (0.709, 0.912)
Continuous (1 h incr)	638/84,597	0.959 (0.859, 1.078)	1097/111,175	0.860 (0.797, 0.933)
Wake time 2				
≤6:30	392/54,600	1.00	770/79,606	1.00
>6:30	246/29,997	0.953 (0.806, 1.125)	327/31,569	0.927 (0.810, 1.218)
Continuous (1 h incr)	638/84,597	0.900 (0.815, 0.995)	1097/111,175	0.978 (0.904, 1.058)
Bedtime 2				
≤22:30	359/54,735	1.00	713/79,562	1.00
>22:30	279/29,862	1.281 (1.090, 1.504)	384/31,613	1.203 (1.057, 1.367)
Continuous (1 h incr)	638/84,597	1.246 (1.121, 1.384)	1097/111,175	1.125 (1.040, 1.218)

1; ORs were adjusted for age, BMI, marital status, education, smoking, alcohol, diabetes, breakfast time or dinner time. 2; ORs were adjusted for age, BMI, marital status, education, smoking, alcohol, diabetes, wake time or bedtime. Abbreviations: CI, confidence interval; OR, odds ratio.

## Data Availability

De-identified data that support the findings of this study are available from the corresponding author upon reasonable request after approval from Southern Medical University and Guangzhou Center for Disease Control and Prevention.

## Supplementary Materials

The following supporting information can be downloaded at: https://www.mdpi.com/article/10.3390/nu18050861/s1, Table S1: The characteristics of excluded participants and its comparison with the included object; Table S2: Association of mealtimes, bed time and wake time with CRC risk by sex. N = 197,507; Table S3: Subgroup analysis of nightly fasting and sleep duration with CRC risk by sex, N = 197,507; Table S4: Associations of nightly fasting and sleep duration with precancerous lesions risk, N = 197,280; Table S5: Associations of mealtimes, bed time and wake time with risk of precancerous lesions. N = 197,280; Table S6: Subgroup analysis of nightly fasting and sleep duration with precancerous lesions by age, N = 197,280; Table S7: Subgroup analysis of nightly fasting and sleep duration with precancerous lesions by sex, N = 197,280; Table S8: Association of mealtimes, bed time and wake time with precancerous lesions risk by age. N = 197,280; Table S9: Association of mealtimes, bed time and wake time with precancerous lesions risk by sex. N = 197,280; Table S10: Association of night fasting duration and sleep duration; Table S11: Subgroup analysis of nightly fasting and sleep duration with CRC risk by age in male, N = 70,798; Table S12: Association of mealtimes, bedtime and wake time with CRC risk by age in male. N = 70,798; Table S13: Subgroup analysis of nightly fasting and sleep duration with CRC risk by age in female, N = 126,709; Table S14: Association of mealtimes, bedtime and wake time with CRC risk by age in female. N = 126,709.

## Abbreviations

The following abbreviations are used in this manuscript:

BMI	Body Mass Index
CRC	Colorectal cancer
DALY	Disability-Adjusted Life Years
FOBT	Fecal Occult Blood Tests
IARC	International Agency for Research on Cancer
ORs	Odds ratios
SD	Standard Deviation
SDI	Socio-Demographic Index
T2D	Type 2 Diabetes
