# Supplementary material for: Exploratory Analysis of Association of Nightly Fasting and Sleep Durations with Colorectal Cancer Risk in Chinese Community-Dwelling Older Adults: A Cross-Sectional Study
Source: Nutrients. 2026 Mar 7;18(5):861. doi: 10.3390/nu18050861 (PMC12987043; doi:10.3390/nu18050861)
Supplement: Supplementary file 1 [file nutrients-18-00861-s001.zip › nutrients-4138765-supplementary.pdf]

Long Nightly Fasting Duration and Shortly Night Sleep Duration as Risk Factors for Colorectal Cancer

Peiqi Huang1, Boyan Zeng1, Sicheng Li1, Ke Zhang1, Chunhao Li1, Yingru Liang2, Bingyu Liuzhang1, Xiaoli Wu1 , Shaohua Xie[3,4], Yan Li 2,*, Bo Zhang1,*

Supplementary Data

Appendix

Supplementary Data 1
Table S1 The characteristics of excluded participants and its comparison with the included object 2
Table S2 Association of mealtimes, bed time and wake time with CRC risk by sex. N=197507. 3
Table S3 Subgroup analysis of nightly fasting and sleep duration with CRC risk by sex, N=197507. 4
Table S4 Associations of nightly fasting and sleep duration with precancerous lesions risk, N=197280. 5
Table S5 Associations of mealtimes, bed time and wake time with risk of precancerous lesions. N=197280. 6
Table S6 Subgroup analysis of nightly fasting and sleep duration with precancerous lesions by age, N=197280. 7
Table S7 Subgroup analysis of nightly fasting and sleep duration with precancerous lesions by sex, N=197280. 8
Table S8 Association of mealtimes, bed time and wake time with precancerous lesions risk by age. N=197280. 9
Table S9 Association of mealtimes, bed time and wake time with precancerous lesions risk by sex. N=197280 10
Table S10 Association of night fasting duration and sleep duration 11
Table S11 Subgroup analysis of nightly fasting and sleep duration with CRC risk by age in male, N=70798. 12
Table S12 Association of mealtimes, bedtime and wake time with CRC risk by age in male. N=70798. 13
Table S13 Subgroup analysis of nightly fasting and sleep duration with CRC risk by age in female, N=126709. 14
Table S14 Association of mealtimes, bedtime and wake time with CRC risk by age in female. N=126709. 15

Table S1 The characteristics of excluded participants and its comparison with the included object

**Variable**	**Included (n = 195772)**	**Excluded (n = 100930)**	**P**
First-stage positive(n%)	17424 (8.9)	8781 (8.7)	0.64
Age, y	61.4 ± 6.4	62.1 ± 6.1	0.37
Sex, %			＜0.001
Male	70798 (35.9)	38858 (38.5)	
Female	126709 (64.1)	62072 (61.5)	
BMI, kg/m2	23.5 ± 3.4	23.5 ± 3.0	0.78
Marital status, %			＜0.001
Married	184633 (94.3)	89516 (87.7)	
Other	11139 (5.7)	12414 (12.3)	
Education, %			<0.001
Illiterate	7445 (3.8)	5854 (5.8)	
Primary school	71425 (36.1)	39564 (39.2)	
Secondary school	103110 (52.2)	46125 (45.7)	
College	14918 (7.5)	8579 (8.5)	
Postgraduate	598 (0.3)	202 (0.2)	
NA	11 (0.0)	606 (0.6)	
Diabetes, %			<0.001
No	170004 (86.1)	3129 (3.1)	
Yes	21568 (10.9)	404 (0.4)	
NA	5935 (3.0)	97397 (96.5)	
Smoking, %			<0.001
Never	165543 (83.8)	3129 (3.1)	
Current	22076 (11.2)	404 (0.4)	
Quit	9860 (5.0)	101 (0.1)	
NA	28 (0.0)	97296 (96.4)	
Alcohol, %			<0.001
Hardly drink	179473 (90.9)	3230 (3.2)	
Yes, but not every month	8544 (4.3)	202 (0.2)	
Yes, but not every week	4661 (2.4)	101(0.1)	
Yes, more than once a day	4778 (2.4)	101 (0.1)	
NA	51 (0.0)	97296 (96.4)	

BMI indicated body mass index. Data were mean ± SD or number (percentage). NA: Not available (non-response).

Table S2 Association of mealtimes, bed time and wake time with CRC risk by sex. N=197507.

Variable	Men		Women	
	N CRC/ non-CRC	OR (95% CI)	N CRC/ non-CRC	OR (95% CI)
Breakfast time 1				
≤ 7:30	534/ 44544	1.00	497/ 80200	1.00
> 7:30	371/ 25349	1.177 (1.028, 1.346)	333/ 45679	1.140 (0.989, 1.312)
Continuous (1h incr)	905/ 69893	1.070 (0.984, 1.161)	830/ 125879	1.058 (0.966, 1.158)
Dinner time 1				
≤ 18:30	543/ 40880	1.00	512/ 73595	1.00
> 18:30	362/ 29013	0.923 (0.805, 1.057)	318/ 52284	0.852 (0.739, 0.812)
Continuous (1h incr)	905/ 69893	0.892 (0.823, 0.977)	830/ 125879	0.904 (0.822, 0.994)
Wake time 2				
≤ 6:30	588/ 45306	1.00	574/ 88900	1.00
> 6:30	317/ 24587	0.985 (0.821, 1.088)	256/ 36979	0.946 (0.810, 1.101)
Continuous (1h incr)	905/ 69893	0.917 (0.794, 1.059)	830/ 125879	0.983 (0.896, 1.078)
Bed time 2				
≤ 22:30	552/ 46350	1.00	520/ 87767	1.00
> 22:30	353/ 23363	1.222 (1.063, 1.403)	310/ 38112	1.259 (1.088, 1.456)
Continuous (1h incr)	905/ 69893	1.171 (1.076, 1.275)	830/ 125879	1.173 (1.068, 1.289)

1; ORs were adjusted for age, BMI, marital status, education, smoking, alcohol, diabetes, breakfast time or dinner time. 2; ORs were adjusted for age, BMI, marital status, education, smoking, alcohol, diabetes, wake time or sleep time. Abbreviations: CI, confidence interval; OR, odds ratio.

Table S3 Subgroup analysis of nightly fasting and sleep duration with CRC risk by sex, N=197507.

Variable	Men		Women	
	N CRC/ non-CRC	OR (95% CI)	N CRC/ non-CRC	OR (95% CI)
Nightly fasting duration 1				
<12	42/ 4071	1 (Ref.)	36/ 7532	1 (Ref.)
12~14	646/ 51552	1.168 (0.864, 1.623)	608/ 93473	1.275 (0.924, 1.189)
≥14	217/ 14270	1.395 (1.009, 1.976)	186/ 24874	1.410 (1.002, 2.054)
Continuous (1h incr)	905/ 69893	1.106 (1.030, 1.187)	830/ 125879	1.083 (1.003, 1.168)
Nightly sleep duration 2				
<9	722/ 52879	1 (Ref.)	660/ 97158	1 (Ref.)
≥9	183/ 17014	0.787 (0.664, 0.929)	170/ 28721	0.867 (0.727, 1.029)
Continuous (1h incr)	905/ 69893	0.855 (0.797, 0.917)	830/ 125879	0.894 (0.828, 0.966)
Dinner-to-bed interval 2				
<4	279/ 25487	1 (Ref.)	269/ 50738	1 (Ref.)
≥4	626/ 44406	1.125 (0.965, 1.313)	561/ 75141	1.257 (1.074, 1.474)
Continuous (1h incr)	905/ 69893	1.135 (1.047, 1.230)	830/ 125879	1.174 (1.073, 1.284)
Wake-to-breakfast interval 2				
<1	216/ 18668	1 (Ref.)	205/ 27809	1 (Ref.)
≥1	689/ 51225	1.128 (0.967, 1.320)	625/ 98070	0.849 (0.724, 1.001)
Continuous (1h incr)	905/ 69893	1.107 (1.009, 1.211)	830/ 125879	0.987 (0,999 1.172)

1; Model adjusted for BMI, marital status, education, smoking, alcohol, diabetes. 2; Model adjusted for fasting time in addition.

Table S4 Associations of nightly fasting and sleep duration with precancerous lesions risk, N=197280.

Variable	Precancerous lesions	
	**N CRC/ non-CRC**	**OR (95% CI)**
Nightly fasting duration 1		
<12	72/ 11603	1 (Ref.)
12~14	1080/ 145025	1.130 (0.896, 1.449)
≥14	356/ 39144	1.338 (1.042, 1.742)
Continuous (1h incr)	1508/ 195772	1.098 (1.039, 1.160)
Nightly Sleep duration 2		
<9	1201/ 150037	1 (Ref.)
≥9	307/ 45735	0.831 (0.730, 0.945)
Continuous (1h incr)	1508/ 195772	0.879 (0.831, 0.929)
Dinner-to-bed interval 2		
<4	476/ 76225	1 (Ref.)
≥4	1032/ 119547	1.191 (1.059, 1.341)
Continuous (1h incr)	1508/ 195772	1.151 (1.080, 1.228)
Wake-to-breakfast interval 2		
<1	365/ 46477	1 (Ref.)
≥1	1143/ 149295	0.969 (0.860, 1.094)
Continuous (1h incr)	1508/ 195772	0.998 (0.964, 1.114)

1; Model adjusted for BMI, marital status, education, smoking, alcohol, diabetes. 2; Model adjusted for fasting time in addition.

Table S5 Associations of mealtimes, bed time and wake time with risk of precancerous lesions. N=197280.

Variable	N CRC/ non-CRC	OR (95% CI)
Breakfast time 1		
≤ 7:30	894/ 124744	1.00
> 7:30	614/ 71028	1.165 (1.049, 1.292)
Continuous (1h incr)	1508/ 195772	1.063 (0.995, 1.134)
Dinner time 1		
≤ 18:30	919/ 114475	1.00
> 18:30	589/ 81297	0.880 (0.792, 0.978)
Continuous (1h incr)	1508/ 195772	0.885 (0.829, 0.950)
Wake time 2		
≤ 6:30	1015/ 134206	1.00
> 6:30	493/ 61566	0.915 (0.817, 1.023)
Continuous (1h incr)	1508/ 195772	0.938 (0.878, 1.002)
Bed time 2		
≤ 22:30	932/ 134297	1.00
> 22:30	576/ 61475	1.235 (1.109, 1.375)
Continuous (1h incr)	1508/ 195772	1.152 (1.076, 1.232)

1; ORs were adjusted for age, sex, BMI, marital status, education, smoking, alcohol, diabetes, breakfast time or dinner time. 2; ORs were adjusted for age, sex, BMI, marital status, education, smoking, alcohol, diabetes, wake time or bed time. Abbreviations: CI, confidence interval; OR, odds ratio.

Table S6 Subgroup analysis of nightly fasting and sleep duration with precancerous lesions by age, N=197280.

Variable	age			
	≦60		>60	
	N CRC/ non-CRC	OR (95% CI)	N CRC/ non-CRC	OR (95% CI)
Nightly fasting duration 1				
<12	30/ 5193	1 (Ref.)	42/ 6410	1 (Ref.)
12~14	419/ 61341	1.113 (0.781, 1.650)	661/ 83684	1.122 (0.831, 1.559)
≥14	133/ 18063	1.137 (0.774, 1.725)	223/ 21081	1.498 (1.084, 2.121)
Continuous (1h incr)	582/ 84597	1.012 (0.928, 1.103)	926/ 111175	1.170 (1.088, 1.258)
Nightly sleep duration 2				
<9	463/ 65331	1 (Ref.)	738/ 84706	1 (Ref.)
≥9	119/ 19266	0.907 (0.733, 1.113)	188/ 26469	0.788 (0.667, 0.927)
Continuous (1h incr)	582/ 84597	0.848 (0.775, 0.929)	926/ 111175	0.898 (0.837, 0.964)
Dinner-to-bed interval 2				
<4	166/ 30377	1 (Ref.)	310/ 45848	1 (Ref.)
≥4	416/ 54220	1.277 (1.051, 1.558)	616/ 65327	1.160 (1.002, 1.346)
Continuous (1h incr)	582/ 84597	1.212 (1.092, 1.346)	926/ 111175	1.134 (1.045, 1.231)
Wake-to-breakfast interval 2				
<1	174/ 21608	1 (Ref.)	191/ 24869	1 (Ref.)
≥1	408/ 62989	0.835 (0.698, 1.003)	735/ 86306	1.061 (0.904, 1.251)
Continuous (1h incr)	582/ 84597	1.031 (0.913, 1.159)	926/ 111175	1.021 (0,930 1.118)

1; Model adjusted for BMI, marital status, education, smoking, alcohol, diabetes. 2; Model adjusted for fasting time in addition.

Table S7 Subgroup analysis of nightly fasting and sleep duration with precancerous lesions by sex, N=197280.

Variable	Men		Women	
	N CRC/ non-CRC	OR (95% CI)	N CRC/ non-CRC	OR (95% CI)
Nightly fasting duration 1				
<12	39/ 4071	1 (Ref.)	33/ 7532	1 (Ref.)
12~14	539/ 51552	1.048 (0.766, 1.478)	541/ 93473	1.233 (0.881, 1.788)
≥14	191/ 14270	1.333 (0.951, 1.916)	165/ 24874	1.371 (0.952, 2.034)
Continuous (1h incr)	769/ 69893	1.115 (1.033, 1.203)	739/ 125879	1.087 (1.003, 1.178)
Nightly sleep duration 2				
<9	609/ 52879	1 (Ref.)	592/ 97158	1 (Ref.)
≥9	160/ 17014	0.813 (0.677, 0.978)	147/ 28721	0.831 (0.688, 0.998)
Continuous (1h incr)	769/ 69893	0.870 (0.807, 0.940)	739/ 125879	0.881 (0.813, 0.956)
Dinner-to-bed interval 2				
<4	241/ 25487	1 (Ref.)	235/ 50738	1 (Ref.)
≥4	528/ 44406	1.088 (0.922, 1.286)	504/ 75141	1.287 (1.089, 1.525)
Continuous (1h incr)	769/ 69893	1.115 (1.022, 1.216)	739/ 125879	1.181 (1.074, 1.299)
Wake-to-breakfast interval 2				
<1	184/ 18668	1 (Ref.)	181/ 27809	1 (Ref.)
≥1	585/ 51225	1.122 (0.950, 1.331)	558/ 98070	0.857 (0.724, 1.020)
Continuous (1h incr)	769/ 69893	1.098 (0.993, 1.211)	739/ 125879	1.003 (0,902 1.114)

1; Model adjusted for BMI, marital status, education, smoking, alcohol, diabetes. 2; Model additionally adjusted for fasting time.

Table S8 Association of mealtimes, bed time and wake time with precancerous lesions risk by age. N=197280.

Variable	age			
	≦60		>60	
	**N CRC/ non-CRC**	**OR (95% CI)**	**N CRC/ non-CRC**	**OR (95% CI)**
Breakfast time 1				
≤ 7:30	335/ 51950	1.00	559/ 72794	1.00
> 7:30	247/ 32647	1.104 (0.935, 1.303)	367/ 38381	1.119 (1.039, 1.358)
Continuous (1h incr)	582/ 84597	0.984 (0.984, 1.161)	926/ 111175	1.109 (1.019, 1.206)
Dinner time 1				
≤ 18:30	320/ 47487	1.00	599/ 66988	1.00
> 18:30	262/ 29997	1.030 (0.878, 1.215)	327/ 31569	0.787 (0.686, 0.903)
Continuous (1h incr)	582/ 84597	0.955 (0.823, 0.977)	926/ 111175	0.843 (0.779, 0.918)
Wake time 2				
≤ 6:30	359/ 54600	1.00	656/ 79606	1.00
> 6:30	223/ 24587	0.950 (0.797, 1.309)	270/ 36979	0.904 (0.779, 1.047)
Continuous (1h incr)	582/ 84597	0.899 (0.810, 0.998)	926/ 111175	0.974 (0.894, 1.061)
Bed time 2				
≤ 22:30	330/ 54735	1.00	602/ 79562	1.00
> 22:30	252/ 29862	1.259 (1.063, 1.490)	324/ 31613	1.210 (1.051, 1.390)
Continuous (1h incr)	582/ 84597	1.221 (1.093, 1.362)	926/ 111175	1.108 (1.018, 1.208)

1; ORs were adjusted for age, BMI, marital status, education, smoking, alcohol, diabetes, breakfast time or dinner time. 2; ORs were adjusted for age, BMI, marital status, education, smoking, alcohol, diabetes, wake time or bed time. Abbreviations: CI, confidence interval; OR, odds ratio.

Table S9 Association of mealtimes, bed time and wake time with precancerous lesions risk by sex. N=197280.

Variable	Men		Women	
	N CRC/ non-CRC	OR (95% CI)	N CRC/ non-CRC	OR (95% CI)
Breakfast time 1				
≤ 7:30	453/ 44544	1.00	441/ 80200	1.00
> 7:30	316/ 25349	1.188 (1.021, 1.367)	298/ 45679	1.146 (0.986, 1.329)
Continuous (1h incr)	769/ 69893	1.063 (0.972, 1.162)	739/ 125879	1.061 (0.963, 1.167)
Dinner time 1				
≤ 18:30	465/ 40880	1.00	454/ 73595	1.00
> 18:30	304/ 29013	0.901 (0.777, 1.044)	285/ 52284	0.857 (0.737, 0.996)
Continuous (1h incr)	769/ 69893	0.877 (0.807, 0.964)	739/ 125879	0.894 (0.810, 0.997)
Wake time 2				
≤ 6:30	453/ 45306	1.00	514/ 88900	1.00
> 6:30	316/ 24587	0.916 (0.783, 1.069)	225/ 36979	0.918 (0.778, 1.079)
Continuous (1h incr)	769/ 69893	0.918 (0.839, 1.238)	739/ 125879	0.966 (0.875, 1.065)
Bed time 2				
≤ 22:30	473/ 46350	1.00	459/ 87767	1.00
> 22:30	296/ 23363	1.187 (1.020, 1.379)	280/ 38112	1.285 (1.100, 1.497)
Continuous (1h incr)	769/ 69893	1.130 (1.031, 1.238)	739/ 125879	1.179 (1.067, 1.303)

1; ORs were adjusted for age, BMI, marital status, education, smoking, alcohol, diabetes, breakfast time or dinner time. 2; ORs were adjusted for age, BMI, marital status, education, smoking, alcohol, diabetes, wake time or bed time. Abbreviations: CI, confidence interval; OR, odds ratio.

Table S10 Association of night fasting duration and sleep duration.

**Variable**	**All**		**Male**		**Female**	
	**Model 1[1]** OR (95% CI)	**Model 2[2]** OR (95% CI)	**Model 1[1]** OR (95% CI)	**Model 2[2]** OR (95% CI)	**Model 1[1]** OR (95% CI)	**Model 2[2]** OR (95% CI)
Fasting duration - Sleep duration	1.176 (1.129, 1.225)	1.143 (1.096, 1.191)	1.201 (1.136, 1.268)	1.163 (1.099, 1.230)	1.145 (1.078, 1.215)	1.118 (1.051, 1.188)
Sleep duration/Fasting duration	0.084 (0.045, 0.158)	0.130 (0.068, 0.246)	0.062 (0.027, 0.148)	0.102 (0.043, 0.245)	0.124 (0.049, 0.314)	0.174（0.068，0.449)

1; Model 1 is not adjusted for any factors. 2; Model 2 adjusted for BMI, marital status, gender, education, smoking, alcohol, diabetes.

Table S11 Subgroup analysis of nightly fasting and sleep duration with CRC risk by age in male, N=70798.

**Variable**	**age**			
	**≦60**		**>60**	
	**N CRC/ non-CRC**	**OR (95% CI)**	**N CRC/ non-CRC**	**OR (95% CI)**
Nightly fasting duration 1				
<12	12/ 1739	1 (Ref.)	30/ 2332	1 (Ref.)
12~14	204/ 19260	1.439 (0.838, 1.731)	442/ 32292	1.025 (0.719, 1.521)
≥14	69/ 5538	1.674 (0.938, 3.264)	148/ 8732	1.251 (1.052, 1.900)
Continuous (1h incr)	285/ 26537	1.051 (0.929, 1.186)	620/ 43356	1.137 (1.041, 1.241)
Nightly sleep duration 2				
<9	232/ 20277	1 (Ref.)	490/ 32602	1 (Ref.)
≥9	53/ 6260	0.768 (0.558, 1.037)	130/ 10754	0.797 (0.650, 0.970)
Continuous (1h incr)	285/ 26537	0.772 (0.683, 0.874)	620/ 43356	0.898 (0.824, 0.978)
Dinner-to-bed interval 2				
<4	75/ 8907	1 (Ref.)	204/ 16580	1 (Ref.)
≥4	210/ 17630	1.259 (1.047, 1.688)	416/ 26776	1.095 (1.014, 1.315)
Continuous (1h incr)	285/ 26537	1.270 (1.100, 1.466)	620/ 43356	1.104 (1.002, 1.217)
Wake-to-breakfast interval 2				
<1	80/ 8007	1 (Ref.)	136/ 10661	1 (Ref.)
≥1	205/ 18530	1.121 (0.865, 1.465)	484/ 32695	1.094 (0.904, 1.332)
Continuous (1h incr)	285/ 26537	1.148 (0.971, 1.344)	620/ 43356	1.056 (0.942, 1.179)

1; Model adjusted for BMI, marital status, education, smoking, alcohol, diabetes. 2; Model adjusted for fasting time in addition.

Table S12 Association of mealtimes, bedtime and wake time with CRC risk by age in male. N=70798.

Variable	age			
	≦60		>60	
	N CRC/ non-CRC	OR (95% CI)	N CRC/ non-CRC	OR (95% CI)
Breakfast time 1				
≤ 7:30	163/ 16741	1.00	371/ 27803	1.00
> 7:30	122/ 9796	1.221 (0.961, 1.546)	249/ 15553	1.157 (0.982, 1.362)
Continuous (1h incr)	285/ 26537	0.995 (0.857, 1.151)	620/43356	1.101 (0.999, 1.224)
Dinner time 1				
≤ 18:30	161/ 14795	1.00	382/ 26085	1.00
> 18:30	124/ 11742	0.948 (0.747, 1.202)	238/ 17271	0.907 (0.768, 1.069)
Continuous (1h incr)	285/ 26537	0.904 (0.793, 1.060)	620/43356	0.904 (0.801, 0.991)
Wake time 2				
≤ 6:30	172/ 16098	1.00	416/ 29208	1.00
> 6:30	113/ 10439	0.867 (0.675, 1.111)	204/ 14148	0.936 (0.782, 1.115)
Continuous (1h incr)	285/ 26537	0.814 (0.705, 1.940)	620/43356	0.965 (0.872, 1.067)
Bedtime 2				
≤ 22:30	152/ 16597	1.00	399/ 29933	1.00
> 22:30	132/ 9940	1.346 (1.059, 1.710)	221/ 13423	1.152 (1.057, 1.364)
Continuous (1h incr)	285/ 26537	1.291 (1.109, 1.502)	620/43356	1.113 (1.005, 1.232)

1; ORs were adjusted for age, BMI, marital status, education, smoking, alcohol, diabetes, breakfast time or dinner time. 2; ORs were adjusted for age, BMI, marital status, education, smoking, alcohol, diabetes, wake time or bedtime. Abbreviations: CI, confidence interval; OR, odds ratio.

Table S13 Subgroup analysis of nightly fasting and sleep duration with CRC risk by age in female, N=126709.

Variable	age			
	≦60		>60	
	N CRC/ non-CRC	OR (95% CI)	N CRC/ non-CRC	OR (95% CI)
Nightly fasting duration 1				
<12	19/ 3454	1 (Ref.)	17/ 4078	1 (Ref.)
12~14	256/ 42081	1.439 (0.838, 1.731)	352/ 51392	1.122 (0.831, 1.559)
≥14	78/ 12525	1.137 (0.774, 1.725)	108/ 12349	1.498 (1.084, 2.121)
Continuous (1h incr)	353/ 58060	0.992 (0.886, 1.112)	477/ 67819	1.161 (1.047, 1.287)
Nightly sleep duration 2				
<9	275/ 45054	1 (Ref.)	385/ 52104	1 (Ref.)
≥9	78/ 13006	1.021 (0.783, 1.318)	92/ 15715	0.767 (0.605, 0.964)
Continuous (1h incr)	353/ 58060	0.902 (0.801, 0.966)	477/ 67819	0.886 (0.802, 0.981)
Dinner-to-bed interval 2				
<4	106/ 21470	1 (Ref.)	163/ 29268	1 (Ref.)
≥4	247/ 36590	1.284 (1.003, 1.653)	314/ 38551	1.254 (1.024, 1.540)
Continuous (1h incr)	353/ 58060	1.187 (1.034, 1.363)	477/ 67819	1.191 (1.058, 1.341)
Wake-to-breakfast interval 2				
<1	107/ 13601	1 (Ref.)	98/ 14208	1 (Ref.)
≥1	246/ 44459	0.712 (0.567, 1.003)	379/ 53611	0.973 (0.779, 1.226)
Continuous (1h incr)	353/ 58060	0.956 (0.814, 1.117)	477/ 67819	0.987 (0.864, 1.123)

1; Model adjusted for BMI, marital status, education, smoking, alcohol, diabetes. 2; Model adjusted for fasting time in addition.

Table S14 Association of mealtimes, bedtime and wake time with CRC risk by age in female. N=126709.

Variable	age			
	≦60		>60	
	N CRC/ non-CRC	OR (95% CI)	N CRC/ non-CRC	OR (95% CI)
Breakfast time 1				
≤ 7:30	201/ 35209	1.00	296/ 44991	1.00
> 7:30	152/ 22851	1.088 (0.878, 1.346)	181/ 22828	1.179 (0.976, 1.421)
Continuous (1h incr)	353/ 58060	1.005 (0.876, 1.151)	477/ 67819	1.097 (0.971, 1.238)
Dinner time 1				
≤ 18:30	189/ 32682	1.00	323/ 40903	1.00
> 18:30	164/ 25368	1.099 (0.889, 1.359)	154/ 26916	0.689 (0.566, 0.836)
Continuous (1h incr)	353/ 58060	1.020 (0.879, 1.200)	477/ 67819	0.832 (0.801, 0.991)
Wake time 2				
≤ 6:30	220/ 38502	1.00	354/ 50398	1.00
> 6:30	133/ 19558	1.016 (0.810, 1.269)	123/ 17421	0.882 (0.712, 1.087)
Continuous (1h incr)	353/ 58060	1.081 (0.854, 1.126)	477/ 67819	0.978 (0.863, 1.108)
Bedtime 2				
≤ 22:30	206/ 38138	1.00	314/ 49629	1.00
> 22:30	147/ 19922	1.221 (1.090, 1.517)	384/ 18190	1.278 (1.049, 1.551)
Continuous (1h incr)	353/ 58060	1.201 (1.037, 1.390)	163/ 67819	1.143 (1.011, 1.293)

1; ORs were adjusted for age, BMI, marital status, education, smoking, alcohol, diabetes, breakfast time or dinner time. 2; ORs were adjusted for age, BMI, marital status, education, smoking, alcohol, diabetes, wake time or bedtime. Abbreviations: CI, confidence interval; OR, odds ratio.
